# How the anisotropy of surface oxide formation influences the transient activity of a surface reaction

**DOI:** 10.1038/s41467-020-20377-9

**Published:** 2021-01-04

**Authors:** P. Winkler, J. Zeininger, Y. Suchorski, M. Stöger-Pollach, P. Zeller, M. Amati, L. Gregoratti, G. Rupprechter

**Affiliations:** 1 Institute of Materials Chemistry, TU Wien, Getreidemarkt 9, 1060 Vienna, Austria; 2 University Service Center for Transmission Electron Microscopy, TU Wien, Wiedner Hauptstraße 8-10, 1040 Vienna, Austria; 3 Elettra–Sincrotrone Trieste S.C.p.A., SS14 - km 163.5 in Area Science Park, 34149 Trieste, Italy

**Keywords:** Catalytic mechanisms, Heterogeneous catalysis, Scanning probe microscopy

## Abstract

Scanning photoelectron microscopy (SPEM) and photoemission electron microscopy (PEEM) allow local surface analysis and visualising ongoing reactions on a µm-scale. These two spatio-temporal imaging methods are applied to polycrystalline Rh, representing a library of well-defined high-Miller-index surface structures. The combination of these techniques enables revealing the anisotropy of surface oxidation, as well as its effect on catalytic hydrogen oxidation. In the present work we observe, using locally-resolved SPEM, structure-sensitive surface oxide formation, which is summarised in an *oxidation map* and quantitatively explained by the novel step density (SDP) and step edge (SEP) parameters. In situ PEEM imaging of ongoing H_2_ oxidation allows a direct comparison of the local reactivity of metallic and oxidised Rh surfaces for the very same different stepped surface structures, demonstrating the effect of Rh surface oxides. Employing the velocity of propagating reaction fronts as indicator of surface reactivity, we observe a high transient activity of Rh surface oxide in H_2_ oxidation. The corresponding *velocity map* reveals the structure-dependence of such activity, representing a direct imaging of a structure-activity relation for plenty of well-defined surface structures within one sample.

## Abstract (web summary)

Surface oxide formation under reaction conditions may change the catalytic activity of a catalyst. Here, the authors explore the effect of atomic structure of Rh surfaces on the surface oxide formation and its influence on catalytic activity in hydrogen oxidation, revealing a high transient activity.

## Introduction

Oxide surfaces are important in many areas of technology, including fuel and energy generation/storage (reforming, syngas, fuel cells, electrolysers and batteries), corrosion, sensors, exhaust gas cleaning, and others^1–5^. Intensive experimental and theoretical research during the last decade has led to the discovery that the transition from the metal to the bulk oxide proceeds via the formation of ultrathin oxide films, which are termed surface oxides^6^. Such surface oxides, with the topmost metal layer sandwiched between two atomic layers of oxygen, can even be considered a new class of materials, as they may exhibit novel unexpected properties^7–9^. The growth of such surface oxides was observed for Rh^7,9–12^, Ru^13^, Pd^6,14,15^ and Pt^15^, which is of academic interest, but also has practical impact on the technologies mentioned.

Much of the atomistic understanding of surface oxidation originates from studies of well-defined model systems in ultrahigh vacuum (UHV)^6–15^. Combined with DFT calculations, these studies identified the atomic structures of surface oxides formed on smooth (low-Miller-index) single crystals of several noble metals^7,11–18^. Usually, surface oxides are explored in time-consuming sequential one-sample-after-another experiments on multiple single crystal samples. However, surface oxidation exhibits anisotropy, so that the local oxidation rates on crystallographically different technologically relevant less-ideal surfaces vary substantially. This important point was not considered until now.

As an alternative approach to sequential experiments, polycrystalline surfaces can be employed as model systems. If the crystallographic orientation of each surface domain is known, polycrystalline surfaces turn into surface structure libraries, since hundreds of domains with different orientations are present on a 1 cm^2^ sample^19^. This concept, in combination with a spatially resolving experimental technique, allows studying surface processes on different surface structures simultaneously^20,21^. In the present work, surface structure libraries are for the first time applied to examine the anisotropy of surface oxidation, exploiting their essential advantage, namely guaranteeing the same oxygen exposures, temperatures and temperature ramps for differently oriented domains. This identity of conditions can hardly be met by traditional sequential experiments. Furthermore, the domains on a polycrystalline sample are not limited to low-Miller-index crystallographic orientations. Typically, several stepped high-index domains are present, resembling rough surfaces or nanoparticles in typical catalytic applications^22^, and these stepped Rh surfaces are in the focus of the present work. Synchrotron-based scanning photoelectron microscopy (SPEM), using a 0.13 µm diameter X-ray microprobe, is used to obtain spatially resolved chemical information and for chemical imaging^23^.

Surface oxides have also received significant attention in heterogeneous catalysis, e.g. in the context of exhaust cleaning or combustion, because depending on reaction conditions an initially metallic surface may be in oxidised or reduced state, significantly changing its activity^10,14^. This shows the strong need for in situ studies of catalytic reactions once surface oxides are involved. For well-defined surface oxides the focus was so far mostly on CO oxidation^13,24,25^. For example, high CO oxidation activity was attributed to a trilayer surface oxide on Rh(111)^25–27^, whereas continued surface oxidation produced an inactive double-trilayer (ORhO-4L^28^) and thicker bulk-like oxides, leading to catalyst deactivation^29^.

To further examine the effect of surface oxides in catalysis, we have chosen the prototypical hydrogen oxidation as a “litmus paper” test. On platinum group metals this surface reaction has been intensively studied since the times of Döbereiner^30^ and Faraday^31^, and even contributed to the introduction of the term “catalysis” by Berzelius^32^. Currently, the societal and technological importance of catalytic H_2_ oxidation increases, due to applications for fuel cells, catalytic heat production, elimination of hydrogen via catalytic recombination and hydrogen sensors^33–36^. On metallic surfaces, numerous atomic scale studies provided deep insights into the reaction mechanism^37–39^, whereas corresponding studies on surface oxides are rare^40,41^. The present work provides such study focusing on the role of the trilayer surface oxide, when large amounts of oxygen are incorporated into the Rh crystal lattice. This is in significant difference to previous studies^21,42,43^ of H_2_ oxidation on metallic Rh, when only small amounts of subsurface oxygen were involved.

**Fig. 1:**
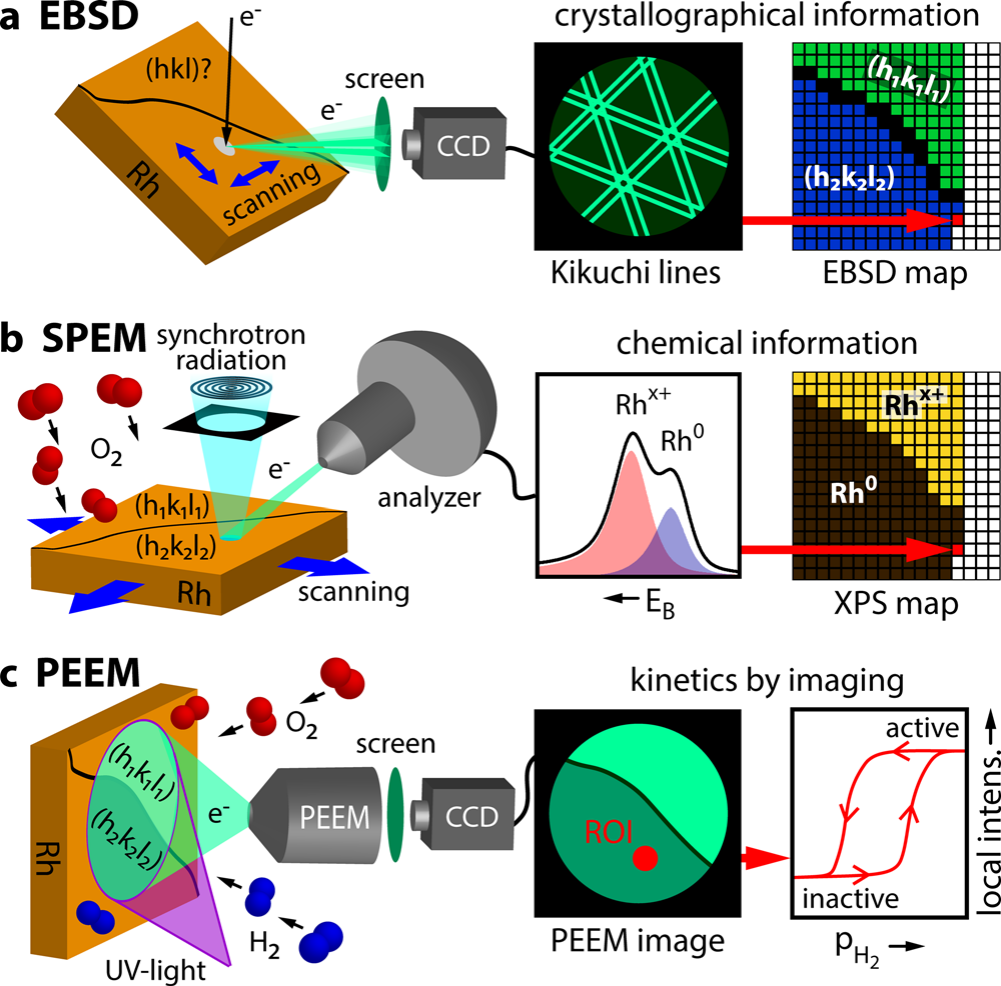
Experimental approach. **a** EBSD: the backscattered electrons of a focused electron beam form Kikuchi lines on a phosphorous screen, enabling determination of the crystallographic orientation of each µm-sized Rh domain; **b** SPEM: an oxidised polycrystalline Rh sample is raster-scanned by a sub-µm-sized X-ray spot, with the emitted photoelectrons providing local XPS spectra. By processing the XPS spectra, the local Rh oxide components can be identified and displayed in a chemical map; **c** PEEM: the ongoing H_2_ oxidation reaction is visualised in situ by PEEM, with the analysis of the local PEEM intensities providing spatially resolved local reaction kinetics.

The experimental approach is illustrated in Fig. 1: The crystallography of each µm-sized Rh(hkl) domain of the polycrystalline surface was characterised by electron backscatter diffraction (EBSD; Fig. 1a, see the Methods section below and the details in the SI). The polycrystalline Rh foil was oxidised in the range of 10^−4^ mbar O_2_ and the oxidised surface studied by locally resolved SPEM, resulting in an “oxidation map” (Fig. 1b). Photoemission electron microscopy (PEEM), with a resolution of ~1 μm, was then employed to visualise the ongoing H_2_ oxidation in situ (Fig. 1c). Using the recently-developed kinetics by imaging approach^44^, the local reaction kinetics of individual Rh(hkl) domains can be extracted from real-time PEEM-video-files (Fig. 1c). An “activity map” was created based on hydrogen front propagation. The very same Rh sample was used in the EBSD, SPEM and PEEM experiments, and even the same field of view was monitored, allowing a direct correlation between the local crystallographic structure and SPEM and PEEM data of local oxidation and reaction kinetics, respectively. This combination of SPEM and PEEM is also promising for application to supported particles, enabling spatio-temporal monitoring of structure, composition and reactivity of technological catalysts, which is mostly performed by e.g. X-Ray, infrared, Raman, magnetic resonance or electron microscopies^45–51^.

In the present work, the anisotropy of surface oxide formation on Rh and its influence on catalytic H_2_ oxidation is studied using the powerful combination of two surface-imaging techniques: SPEM and PEEM. SPEM, yielding spatially resolved chemical information, reveals the correlation of initial surface structures and the formation of surface oxides. PEEM, in turn, is used to image H_2_ oxidation reaction kinetics of the different highly stepped surface structures. This provides us immediate insight into the role of Rh surface oxides in catalytic H_2_ oxidation. The velocities of reaction fronts propagating across the surface serve as an indicator for surface reactivity, revealing a high transient activity of Rh surface oxide.

## Results and discussion

### Mapping anisotropic Rh surface oxidation

The surface oxidation of Rh and the structure of the resulting surface oxides has been previously studied via various experimental methods, focusing on well-defined Rh single crystal surfaces of mainly low-Miller-index orientations. Techniques such as low energy electron diffraction (LEED), scanning tunnelling microscopy (STM), surface X-ray diffraction (SXRD) and X-ray photoelectron spectroscopy (XPS)^7,11,12,52^ were applied. The acquired XPS spectra and STM/SXRD-derived atomic structures were corroborated and explained by ab initio calculations^7,11,12^. Herein, we present the first spatially resolved comparative study of the oxidation of high-Miller-index Rh surfaces.

**Fig. 2:**
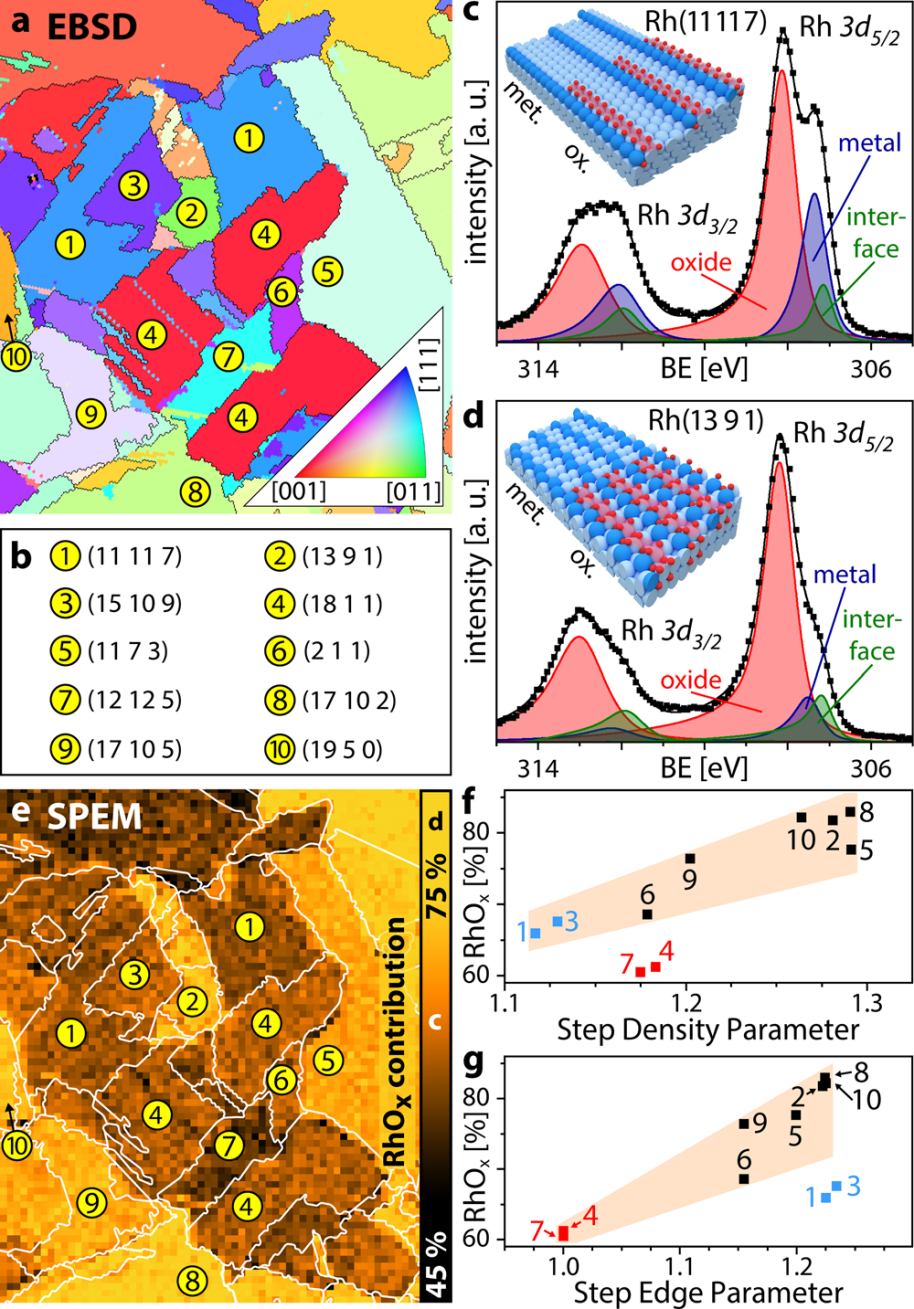
Mapping anisotropic Rh surface oxidation. **a** EBSD image of the polycrystalline Rh foil (512 × 600 µm); **b** crystallographic orientations (Miller indices) of the Rh(hkl) domains indicated in **a** and **e**; **c**, **d** examples of local XPS spectra and ball models of the metallic and oxidised Rh(11 11 7) and Rh(13 9 1) domains, respectively. Squares: measured values; solid lines: sum of the deconvoluted components; **e** *oxidation map* of the region displayed in **a** after oxidation in O_2_ (*T* = 623 K, pO_2_ = 2.5 × 10^−4^ mbar, *t* = 90 min), with the right edge of the map displaying the colour scale for the RhO_x_ contribution; **f**, **g** show the RhO_x_ contribution versus the step density parameter (SDP) and step edge parameter (SEP), respectively (see SI for a detailed description of SDP and SEP). Squares: measured values (see text for the highlighted data points); shaded areas serve as a guide for the eye.

Figure 2a shows the EBSD map of a polycrystalline Rh foil, consisting of well-defined Rh(hkl) domains, as listed in Fig. 2b. After oxidation at *T* = 623 K, pO_2_ = 2.5 × 10^−4^ mbar, *t* = 90 min, i.e., under conditions similar to those reported for trilayer oxide growth on single crystals^7,11,12^, local XPS spectra (exemplarily shown in Fig. 2c, d) were measured for a 0.13 µm diameter spot on each 8 × 8 µm^2^ sample area by SPEM. Due to the chosen photon energy of ~650 eV, the kinetic energy of the Rh *3d* photoelectrons was in the range of 340–350 eV. In combination with the grazing collection angle, this provides high surface sensitivity with a probing depth of ~1.5 nm. The deconvolution of the measured Rh *3d* spectra allows determining the metallic, oxidic and interfacial (i.e. the Rh atoms at the interface between the Rh bulk and the trilayer oxide) contributions for each Rh(hkl) domain in the field of view. The resulting peak positions are in quantitative agreement with XPS results for Rh surface oxides formed on low-Miller-index surfaces^7,11,12^, but differ from thicker bulk-like oxides^10^ and metallic Rh with adsorbed oxygen^7^.

Figure 2c shows the spectrum deconvolution for the Rh(11 11 7) domain (position 1 in Fig. 2a) with 26%, 64 and 10% contributions by metallic, oxidic and interface Rh, respectively (see the SI for details). Corresponding results for the Rh(13 9 1) domain (position 2 in Fig. 2a) with 10%, 78% and 12% of Rh, RhO_x_ and interfacial Rh, respectively, are shown in Fig. 2d. Note that the trilayer oxide structures formed on Rh(111), Rh(100) and Rh(110) exhibit the same hexagonal symmetry, differing slightly by the lattice constant (3.02 Å, 3.08 Å and 3.04 Å, respectively^7,11,12^), while their Rh *3d* peak positions are identical^7,11,12^. This is also the case for the present Rh(11 11 7) surface, consisting of (111) fcc terraces, and Rh(13 9 1), consisting of (110) fcc terraces.

The XPS results for each Rh(hkl) domain in the field of view (512 × 600 µm^2^, Fig. 2a) are summarised as a chemical map of the surface oxide contribution (Fig. 2e), directly illustrating the anisotropy of the surface oxidation of different stepped Rh(hkl) surfaces. As the formed surface oxides are structurally nearly identical (see SI), the differences in the oxide contributions may thus result from different extent of oxide coverage on the metallic substrate. In this respect, we refer to recent STM results, which revealed a stripe-like growth of the Rh surface oxide: patches of the O-Rh–O trilayer were first formed along the step edges, before extending to the Rh(111) terraces^53^. Kinetic limitations of stripe-like growth, predicted by DFT, result in only partial coverage of wider terraces by surface oxide (see schematic models in Fig. 2c, d). Thus, domains differing in the width of their terraces will be covered by surface oxide to a different extent, leading to differing metal/oxide area ratios. Consequently, the RhO_x_ contribution to the XPS signal depends on the step density. In addition, the atomic structure of the step edges (e.g. the number of kinks) modifies the kinetic barriers for surface oxide formation^54^. This will result in differing shapes of the oxide stripes, again varying the RhO_x_ contribution to the XPS signal. To analyse the dependence of surface oxide formation on a particular surface orientation (structure), both the step density and step edge configurations must be considered. Thus, we have developed a numerical description of these two relevant aspects, introducing the dimensionless step density parameter (SDP) and step edge parameter (SEP). Both parameters are based on the surface free energy in relation to the smooth close-packed (111) fcc surface. The SDP, characterising the density of atomic steps, is calculated from a model surface having the same terrace structure and width as the considered surface, but simplified non-kinked step edges. The SEP, in turn, is calculated from a model surface formed by the step edge rows of the considered surface and thus describes the particular atomic configuration of the step edges. Further details on the calculation of SDP and SEP are given in the SI.

Plots of the surface oxide contribution versus SDP and SEP are shown in Fig. 2f, g, respectively, displaying a clear trend of higher RhO_x_ extent for increasing SDP and SEP. A qualitative trend of stronger surface oxidation for higher atomic roughness (due to steps and kinks) has been reported in literature^53^, but, to our knowledge, the present SDP/SEP evaluation is the first to address both factors quantitatively. Even the apparent outliers (i.e. data points outside of the shaded areas in Fig. 2f, g) can be explained by this concept: the lower RhO_x_ contribution of Rh(18 1 1) and Rh(12 12 5) (red squares, positions 4 and 7 in Fig. 2f) is due to their position on the SEP axis (see Fig. 2g). Similarly, Rh(11 11 7) and Rh(15 10 9) (blue squares, positions 1 and 3 in Fig. 2g) have lower RhO_x_ contribution due to their position on the SDP axis (see Fig. 2f). For more details concerning the interrelation of SDP and SEP see the SI.

### Impact of surface oxidation anisotropy on catalytic H_2_ oxidation

**Fig. 3:**
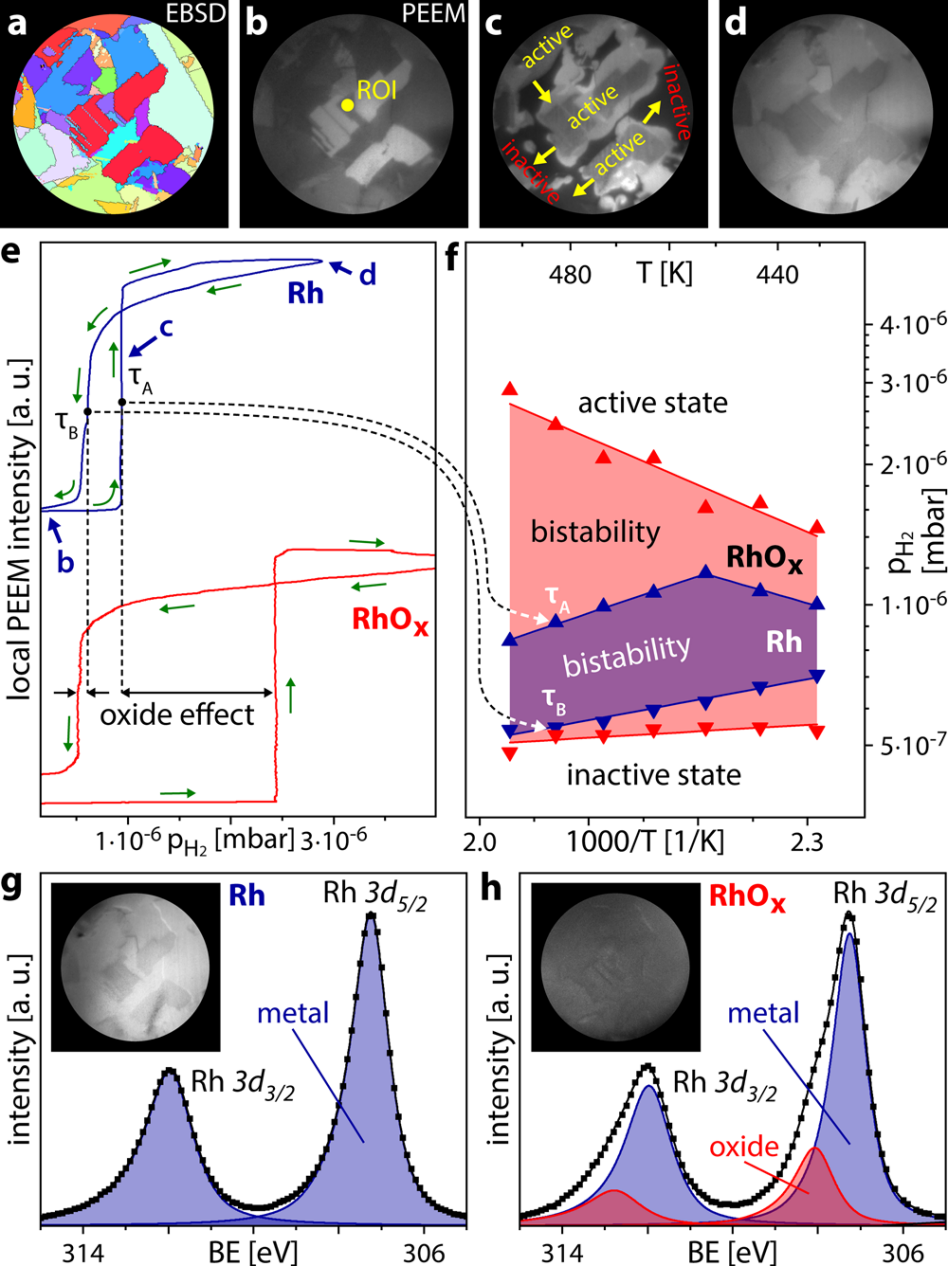
Imaging catalytic H_2_ oxidation on metallic and oxidised Rh. **a** EBSD map of the chosen Rh sample region (diameter 550 µm, cf. Fig. 2a); **b** the same region imaged by PEEM (oxygen-covered metallic Rh surface), the contrast reflects the work function differences between different Rh(hkl) domains; **c** in situ PEEM image of an ongoing kinetic transition during H_2_ oxidation, arrows show directions of propagating reaction fronts, bright areas correspond to the catalytically active surface; **d** PEEM image of the catalytically active steady-state resulting from the transition depicted in **c**; **e** hysteresis curves registered by processing the local PEEM intensity of the ROI shown in **b** during cyclewise variation of the H_2_/O_2_ pressure ratio from 0.1 to 6.5 at constant O_2_ pressure of 7.7 × 10^−7^ mbar and temperature of 483 K. The upper and lower hysteresis curves correspond to the metallic and oxidised Rh surface, respectively; **f** kinetic phase diagrams for H_2_ oxidation on the metallic (shaded blue) and oxidised (shaded red) Rh surface; **g**, **h** Rh *3d* XPS spectra obtained by lab-XPS (excitation energy 1253.6 eV), for the metallic and oxidised Rh surface, respectively. Squares: measured values; solid lines: sum of the deconvoluted components. The insets show corresponding PEEM images.

In the following, catalytic H_2_ oxidation is used as a probe to indicate the effect of the surface oxidation anisotropy. For catalytic H_2_ oxidation on Rh, both reactants need to adsorb to the catalyst surface before the reaction can take place (Langmuir-Hinshelwood mechanism)^42,55^. At low pH_2_/pO_2_ ratio the reaction system is in an inactive steady-state (adsorbed oxygen blocks the adsorption of hydrogen). Increasing the pH_2_/pO_2_ ratio at constant temperature lets the system switch to an active steady-state at a certain pH_2_/pO_2_ ratio. This, as well as the reverse switch, occur via a kinetic transition, a process similar to a phase transition in equilibrium thermodynamics (whereas kinetic transitions in catalysis are topics of non-equilibrium thermodynamics^56^). Such kinetic transitions can be visualised by PEEM, as the brightness of the PEEM image results from the photoemission yield governed by the work function of the imaged surface, which, in turn, depends on the surface coverage. For example, the inactive oxygen-covered Rh surface has a higher work function than the adsorbate free Rh surface and thus appears dark in PEEM contrast. Vice versa, the catalytically active surface with low H_ads_ and O_ads_ coverage is characterised by lower work function and thus appears bright. These image contrast differences are the basis of the kinetics by imaging approach in H_2_ oxidation on Rh^21,57^ and allow extracting the local kinetic information from in situ recorded PEEM video-sequences (for details see the SI). In the present studies, the same Rh sample as in the SPEM experiments was used. The PEEM chamber was filled with gaseous O_2_ and H_2_ in the 10^−6^ mbar pressure range and operated like a flow reactor, and the reaction on the Rh sample was visualised and video-recorded in real time, as summarised in Fig. 3. The fields of view in the SPEM and PEEM experiments are almost completely overlapping (cf. EBSD maps in Figs. 2a and 3a, a comparison is shown in the SI).

Catalytic experiments both on metallic and oxidised Rh always started from the inactive O-covered state (Fig. 3b). Increasing pH_2_ at constant pO_2_ and T leads to a kinetic transition to the active state at a particular pH_2_ value *τ*_A_. The transition was accompanied by H_ads_ fronts spreading over the entire field of view (Fig. 3c), and resulted in the catalytically active nearly adsorbate-free state (Fig. 3d). Subsequently decreasing the H_2_ partial pressure caused the reverse kinetic transition at a pH_2_ value *τ*_B_, with *τ*_B_ being significantly lower than *τ*_A_.

From the recorded video-sequences, the local PEEM image intensity can be read out for regions of interest (ROIs) placed at arbitrarily chosen locations on the surface. As an example for metallic Rh, the local intensity evaluated within the ROI marked in Fig. 3b is plotted versus the H_2_ partial pressure (*T* = 483 K, pO_2_ = 7.7 × 10^−7^ mbar), yielding the blue trace in Fig. 3e. The curve shows a pronounced hysteresis between the kinetic transition points *τ*_A_ and *τ*_B_, indicating a bistability of the reaction: between *τ*_A_ and *τ*_B_, the system can exist either in its active or inactive state, depending solely on the sample prehistory^42,57^.

To examine the effect of the Rh trilayer oxide on catalytic H_2_ oxidation, the Rh sample was oxidised at the same conditions as described for the SPEM experiments (Fig. 2). To ensure the same oxidation state of the Rh sample before each catalytic experiment, a laboratory XPS system, directly connected to the PEEM chamber, was used. Exemplary Rh *3d* spectra and corresponding PEEM images of the clean and oxidised surfaces are shown in Fig. 3g, h, respectively. In comparison to the synchrotron SPEM (photon energy 650 eV, take-off angle 60°, probing depth ~1.5 nm) the lab-XPS (photon energy 1253.6 eV, take-off angle 0°), provides reduced surface sensitivity (due to the higher probing depth of ~5 nm) and energy resolution (0.8 eV versus 0.3 eV in the SPEM experiments). This necessitates a different deconvolution procedure and hampers the discrimination of the interface and metallic components, but the surface oxide component relevant in the present study can still be reliably discerned.

After Rh surface oxidation, the H_2_ oxidation reaction was again monitored by PEEM and the local image intensity was evaluated. The red trace in Fig. 3e shows the exemplary result for the same ROI as for the metallic surface. Again, a hysteresis was observed, but with much wider hysteresis loop for the oxidised Rh surface than for metallic Rh: *τ*_A_ is shifted to significantly higher pH_2_, while *τ*_B_ is shifted to slightly smaller pH_2_. Such experiments were repeated at different temperatures in the range of 433–493 K and the transition points are summarised in a kinetic phase diagram, shown in Fig. 3f, both for the metallic (shaded blue) and the oxidised (shaded red) Rh surface. The same trends were observed in the whole temperature region studied, i.e. the region of bistability is generally wider for oxidised Rh.

To rationalise the different catalytic behaviour of metallic and oxidised Rh we refer to recent STM and high-resolution XPS studies, complemented by calculations performed via the Johnson-Mehl-Avrami-Kolmogoroff (JMAK) theory^58^. As shown in that study, H_2_ hardly adsorbs on Rh trilayer oxide and the reduction almost exclusively started at steps providing sites for dissociative H_2_ adsorption. In our experiments, particularly the step edges were covered by surface oxide (see the insets in Fig. 2c, d), so significantly higher H_2_ pressures (*τ*_A_ values) were needed to initiate dissociative H_2_ adsorption, e.g. at a step edge or a terrace defect. For the reverse kinetic transition at *τ*_B_, the relevant sites on the step edges were already oxide-free. Accordingly, the kinetic transitions to the inactive state occurred at a H_2_ pressures *τ*_B_ close to that of the metallic Rh surface. Therefore, the entire bistability region in the phase diagram for oxidised Rh in Fig. 3f is wider than that for metallic Rh. Clearly, exposures to reducing conditions may partially reduce the Rh oxide surface and modify the hysteresis loop (see the SI for details).

Due to the fast diffusion of atomic hydrogen on metallic and oxidic Rh surfaces, also across grain boundaries, the reaction is not confined to particular domains (in contrary to CO oxidation on Pt and Pd^20^). As a result, kinetic transitions initiated at a specific location quickly spilled-over to neighbouring domains and finally across the entire sample. At first glance, this seems to wipe out the main advantage of a surface structure library, namely the access to structure-sensitivity. However, a detailed view on exploiting the parallel imaging principle of PEEM reveals an interesting additional feature: PEEM provides simultaneous monitoring of the front propagation on different Rh(hkl) domains, allowing a direct comparison of the front velocities and their association with crystallographic orientations of particular Rh(hkl) domains.

**Fig. 4:**
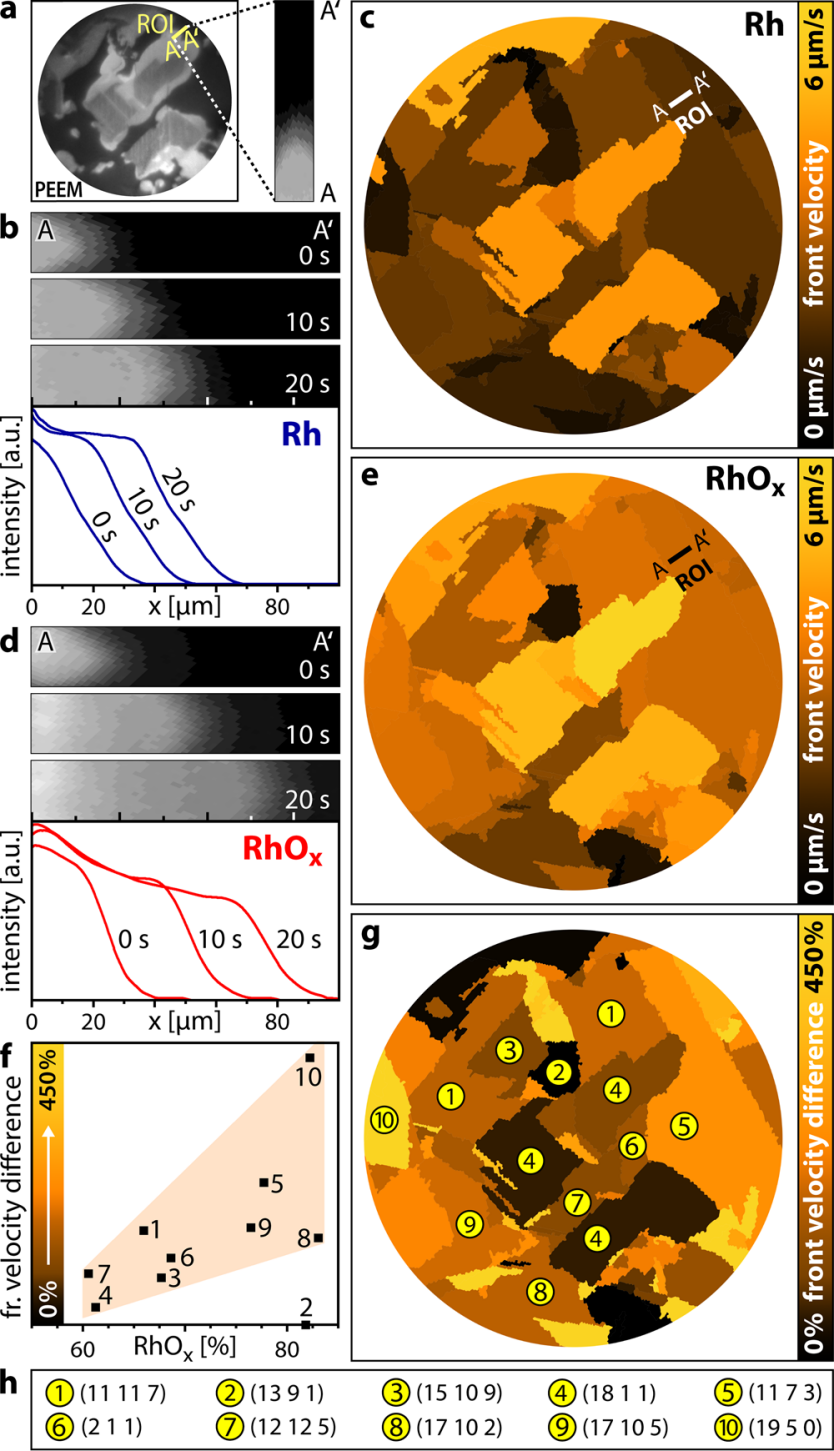
Mapping reaction front propagation in H_2_ oxidation on Rh. **a** in situ PEEM image during an ongoing kinetic transition from the inactive to the active steady state on metallic Rh (*T* = 483 K, pO_2_ = 7.7 × 10^−7^ mbar, pH_2_ = 9.2 × 10^−7^ mbar) and a magnified ROI (15 × 50 µm^2^); **b** consecutive PEEM snapshots (10 s interval) for the ROI marked in **a** and respective intensity profiles for the front propagation on metallic Rh; **c** the corresponding *front velocity map* of metallic Rh, the colour scale is placed on the right; **d** and **e** are analogous to **b** and **c**, but for the oxidised Rh surface (*T* = 483 K, pO_2_ = 7.7 × 10^−7^ mbar, pH_2_ = 2.4 × 10^−6^ mbar); **f** front velocity difference for selected domains of the oxidised Rh surface relative to metallic Rh. Squares: measured values; the shaded area illustrates the trend; **g** *velocity difference map*, the colour scale (numerically corresponding to the ordinate in **f**), is placed on the right; **h** crystallographic orientations (Miller indices) of the Rh(hkl) domains indicated in **g**.

This is illustrated in Fig. 4a–c, analysing the reaction front propagation on metallic Rh during the kinetic transition from the inactive to the active steady state (transition *τ*_A_ in Fig. 3e, pH_2_/pO_2_ = 1.2). Exemplarily, a rectangular ROI (15 × 50 µm²) is placed on the Rh(11 7 3) domain (Fig. 4a), with the position of the reaction front being clearly visible as dividing line between the active (bright) and inactive (dark) Rh surface. The local front velocity was evaluated from the time dependence of the front position, as shown in three exemplary cut-outs of PEEM snapshots (10 s interval) and corresponding intensity profiles in Fig. 4b.

For the different domains, a *front velocity map* can then be constructed, with the values averaged over the particular µm-sized Rh(hkl) domains (Fig. 4c). Such *velocity map* clearly shows a pronounced structure-sensitivity of the front propagation velocity ranging between 0.77 µm/s for Rh(19 5 0) and 4.40 µm/s for Rh(18 1 1).

To study how the surface oxide affected the reaction front propagation, the analogous kinetic transition was analysed for the oxidised Rh(hkl) surfaces at the same *T* and pO_2_. Due to the presence of the surface oxide, the transition occurred at somewhat higher hydrogen pressure of 2.4 × 10^−6^ mbar (Fig. 3e, pH_2_/pO_2_ = 3.1). The exemplary intensity profiles and the *front velocity map* are depicted in Fig. 4d, e, respectively. We note that also for the oxidised Rh(hkl) surfaces the structure-sensitivity of the front propagation is maintained. Comparing the results for metallic and oxidised surfaces reveals that the front propagation velocity is generally higher for the oxidised surfaces.

At first, one is tempted to attribute this to the higher pH_2_ required to initiate the kinetic transition on the oxidised surface. However, the different degree of increase in front velocity for differing Rh(hkl) domains indicates that this is not the main reason (Fig. 4e is not simply a brighter version of Fig. 4c). Indeed, previous experiments on surface oxidised Rh(110) reported an easier reduction of the oxide trilayer (especially of on-surface oxygen), whereas reduction of chemisorbed oxygen on Rh metal required a 40K higher temperature^59^. For the reaction on the oxidised Rh sample, in the initial inactive state, RhO_x_ stripes along the step edges alternate with stripes of O_ads_-covered metallic Rh terraces (Fig. 2c, d). The hydrogen front then propagates significantly faster over the surface oxide than over the O_ads_-covered metallic surface. As a result, the higher the extent of the surface oxide is, the faster the reaction front propagates over the domains.

To prove this relation between the extent of surface oxidation (as determined by SPEM) and the front propagation velocity (as determined by PEEM), ten different Rh(hkl) domains were analysed in detail, indeed revealing a correlation between the RhO_x_ contribution and the front velocity (Fig. 4f). The only exception is Rh(13 9 1) (number 2), which can be explained by the strong (110) component of its surface structure: the reaction front acceleration on the oxide is compensated by plenty atomic furrows on the terraces.

The results on the front propagation are summarised in a *velocity difference map* (Fig. 4g), depicting the structure-dependence of the velocity difference between Rh oxide and metal. We note that the *velocity difference map* strongly resembles the *oxidation map* in Fig. 2e: domains with a higher surface oxide contribution exhibit a stronger effect on the front propagation. This once more emphasises the intrinsic relation between the presence and extent of Rh surface oxide and the reaction front propagation, the latter transmitting kinetic transitions between the catalytically active and inactive states of the catalyst surface^60,61^. Compared to O_ads_ on metallic Rh, the oxygen atoms of the trilayer oxide have lower average binding energy, resulting from the distortion of the Rh lattice upon oxygen incorporation into the subsurface^62,63^. As a result, the surface oxygen atoms can be removed more easily, effectively lowering the activation barrier for the oxidation reaction (similar as calculated for CO oxidation^17^). The fronts move faster and the *front velocity map* directly reflects the increased activity. After RhO_x_ has been reduced, the active steady state in H_2_ oxidation is metallic Rh with low adsorbate coverage. However, the simultaneous real-time collection of data for different crystallographic orientations allows insight even into temporary catalytic phases, i.e. provides a direct visualisation of the transient surface oxide activity.

In the present study we demonstrate the combined power of two surface-imaging techniques, when applied to the very same sample regions: scanning photoelectron microscopy (SPEM) allows local XPS chemical analysis on a sub-µm-scale, while photoemission electron microscopy (PEEM), based on the parallel imaging principle, allows simultaneously monitoring reactions on different regions. When applied to a polycrystalline sample, representing a library of different well-defined surface structures, this specific combination enables a direct correlation between initial surface structures, the formation of surface oxides, and their resulting catalytic properties. SPEM revealed the effect of the step and kink density of different Rh(hkl) domains on the extent of Rh surface oxide formation, with the step density (SDP) and step edge (SEP) parameters enabling quantitative correlations.

In turn, PEEM imaged the H_2_ oxidation reaction kinetics of different highly stepped surface structures, both for metallic and oxidised surfaces of the very same sample region. The parallel imaging principle of PEEM enabled us to simultaneously register the local reactivity of dozens of domains with differing surface structures. This allowed mapping of reactivity on a µm-scale, providing immediate insight into the role of surface structure and surface oxidation, and to address the role of Rh surface oxides in catalytic H_2_ oxidation. Using the velocity of reaction fronts propagating across the surface during kinetic transitions as indicators of surface reactivity, a high transient activity of Rh surface oxide was detected. Since the local activity enhancement appeared to be structure-dependent, one can consider this a direct imaging of a structure-activity relation for plenty of well-defined structures combined within one sample.

## Methods

### Preparation and characterisation of the Rh sample

A polished Rh foil (10 × 12 mm^2^, 0.2 mm thickness, 99.99% purity, MaTecK) was used as polycrystalline Rh sample. The sample was cleaned in UHV by repeated cycles of Ar^+^ ion sputtering at 1 keV at 300 K, annealing to 1073–1173 K and consecutive chemical treatment in oxygen (pO_2_ = 5 × 10^−7^ mbar at 773 K) and hydrogen (pH_2_ = 5 × 10^−6^ mbar at 773 K). Cleanliness of the sample was verified before each experiment by SPEM or lab-XPS. The foil temperature was measured by a Type K thermocouple spot-welded to its front. Characterisation of the sample crystallography was performed by electron backscatter diffraction (EBSD), providing the crystallographic orientation of each µm-sized domain by scanning the sample surface with a focused electron beam and recording the diffraction patterns generated by the backscattered electrons. EBSD measurements were performed in a field emission scanning electron microscope (FEI Quanta 200 F) using standard EBSD conditions and evaluation procedures^64^, more details are given in the SI.

### Surface oxidation of Rh

Experiments on the surface oxidation of Rh were performed at the ESCA Microscopy beamline of the Elettra synchrotron facility, which has been described in detail elsewhere^65^. Summarising, the end station consists of three UHV sub-chambers: the sample is introduced to the system via a fast-entry load lock attached to the first chamber. Using magnetic transfer arms and wobble sticks, the sample can be transferred in UHV to a preparation chamber, which is equipped with facilities for Ar^+^ ion sputtering, annealing, high purity gas supply (H_2_: 99.999%, O_2_: 99.999%) and Auger Electron Spectroscopy (AES) for checking sample composition and cleanliness. Afterwards, the sample is transferred in UHV to the SPEM chamber, hosting the zone plate optical system, which provides the X-ray microprobe, a piezo specimen positioning and scanning system and a hemispherical energy analyser equipped with a 48-channel detector.

The SPEM was operated with a lateral resolution of 0.13 µm in the micro-spectroscopy mode and data recorded for a 0.13-µm spot on each 8 × 8 µm² sample area in the spectro-microscopy mode with 0.3 eV energy resolution^66^. Due to the setup geometry, electrons emitted at an angle of 60° to the surface normal were registered. The surfaces were oxidised in the preparation chamber and the sample immediately transferred to the SPEM chamber. Spectra were taken at a photon energy of 650.9 eV and measured in a background pressure of 1 × 10^−7^ mbar O_2_ at 300 K, in order to prevent oxide reduction by residual gas (CO or H_2_). Calibration of the SPEM magnification was achieved by comparing the Rh *3d* elemental maps, displaying topographic contrast, with optical micrographs of the polycrystalline Rh sample.

### Catalytic H_2_ oxidation on metallic and oxidised Rh

Experiments of catalytic H_2_ oxidation on metallic and oxidised Rh surfaces were conducted in a UHV setup consisting of separate chambers for PEEM and XPS, interconnected by a sample transfer tunnel. The PEEM chamber is equipped with sample cleaning facilities, a PEEM (Staib Instruments PEEM 150), a deuterium discharge UV lamp (Heraeus D200F, photon energy ~6.5 eV), a quadrupole mass spectrometer (MKS e-Vision 2) and a high purity gas supply system (H_2_: 99.9995%, O_2_: 99.9999%). In addition to similar sample cleaning and gas dosing facilities, the XPS chamber is equipped with a twin anode X-ray source (Specs XR-50) and a hemispherical energy analyser (Specs Phoibos 100).

The ongoing H_2_ oxidation reaction was visualised in situ by PEEM and the images were recorded by a high-speed CCD camera (Hamamatsu C11440-42U30). Calibration of the PEEM magnification was done by comparison of PEEM images with optical micrographs of the polycrystalline Rh sample. Apart from assessing the cleanliness of the sample, XPS was mainly used to verify the oxidation state of the Rh sample before performing PEEM experiments on the oxidised Rh surfaces. The XPS spectra were acquired from the centre of the sample (circular area of 100 µm in diameter), using Mg K_α_ X-ray radiation, with the energy analyser oriented perpendicular to the sample surface (take-off angle 0°).

### Reporting summary

Further information on research design is available in the Nature Research Reporting Summary linked to this article.

## Supplementary information

Supplementary Information

Reporting Summary

## Data Availability

The data that support the findings of this study are available from the corresponding author upon reasonable request.
