# Supplementary material for: How the anisotropy of surface oxide formation influences the transient activity of a surface reaction
Source: Nat Commun. 2021 Jan 4;12:69. doi: 10.1038/s41467-020-20377-9 (PMC7782819; doi:10.1038/s41467-020-20377-9)
Supplement: Supplementary file 1 — Supplementary Information [file 41467_2020_20377_MOESM1_ESM.pdf]

How the anisotropy of surface oxide formation influences the transient activity of a surface reaction

P. Winkler[1], J. Zeininger[1], Y. Suchorski[1], M. Stöger-Pollach[2], P. Zeller[3], M. Amati[3], L. Gregoratti[3], and G. Rupprechter[1*]

[1]*Institute of Materials Chemistry, TU Wien, Getreidemarkt 9, 1060 Vienna, Austria*
[2]*University Service Center for Transmission Electron Microscopy, TU Wien, Wiedner Hauptstraße 8-10, 1040 Vienna, Austria*
[3]*Elettra–Sincrotrone Trieste S.C.p.A., SS14 - km 163.5 in Area Science Park, 34149 Trieste, Italy*

Supplementary Information

Supplementary Note 1: EBSD characterisation of the Rh sample

In order to study processes such as adsorption, surface oxidation and reaction on individual µm-sized domains of the polycrystalline Rh foil and, particularly, to uncover the anisotropy of these processes, the crystallographic orientations of particular Rh(hkl) domains need to be determined first. In the present study, this was carried out by electron backscatter diffraction (EBSD). EBSD is a well-established crystallographic microstructural characterisation technique based on scanning electron microscopy. In EBSD experiments, backscattered electrons of an electron beam, focused on a particular region of a sample, form a backscatter Kikuchi diffraction pattern (BKDP), which corresponds to each of the diffracting crystal lattice planes [1, 2]. EBSD is commonly used in studies of crystalline or polycrystalline materials, e.g. in metallurgy, to understand recrystallisation and grain-growth processes [3, 4]. EBSD was also successfully applied to polycrystalline foils, e.g. to correlate microstructure with electrochemical properties [5] or with catalytic properties of platinum group metals [6, 7, 8]. In the present study, the EBSD measurements were performed by a field emission scanning electron microscope (FEI Quanta 200F) using standard EBSD conditions and evaluation procedures [9].

The corresponding results are shown as a color-coded map in Supplementary Fig. 1a, the dashed lines indicate the regions studied in the SPEM (rectangular region) and PEEM (circular region) experiments. In order to quantify the influence of structural features (e.g. atomic steps and kinks) a set of ten domains (indicated by numbers in Supplementary Fig. 1a) was selected from the studied region, aiming at a resulting broad variety of surface structures. In Supplementary Fig. 1b these domains and their corresponding Miller indices are listed and marked on the colour key (the latter used for colouring the domains in the EBSD map in Supplementary Fig. 1a). Atomic ball models of the crystallographically different stepped Rh surfaces are given in Supplementary Fig. 1c.

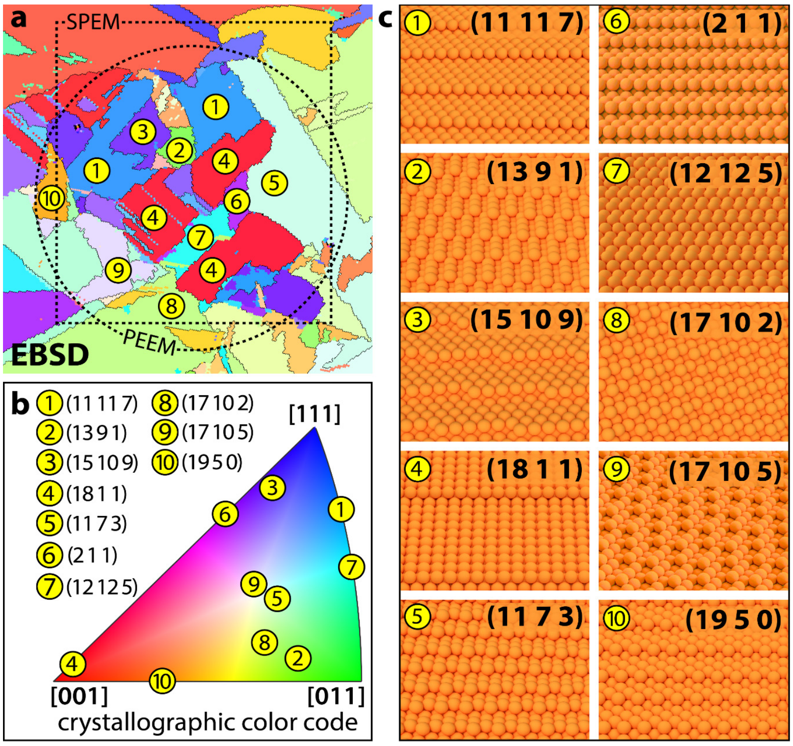

Supplementary Figure 1. Determination of the crystallographic orientation and surface structure of individual domains of a polycrystalline Rh foil: (a) EBSD color-coded map of the studied region; the areas imaged by SPEM and PEEM are indicated by dashed lines; (b) Miller indices of ten domains indicated in (a), selected in order to study structural effects, and their location on the EBSD colour key; (c) atomic ball models of the selected set of domains shown in (a) and (b).

Supplementary Note 2: Surface oxides of Rh

The surface oxidation of rhodium has been studied using various techniques such as X-ray photoelectron spectroscopy (XPS), surface X-ray diffraction (SXRD) and scanning tunnelling microscopy (STM), in combination with ab-initio calculations for mostly simple single crystal surfaces such as (100) [10], (111) [11, 12] and (110) [13]. These studies revealed that at the used oxygen partial pressures ($10^{-5} < p_{O_2} < 10^{-3}$ mbar) the formation of a trilayer surface oxide takes place, while the formation of thicker, bulk-like oxide is kinetically hindered. It is important to note that the atomic structure of the surface oxide is similar for all the mentioned low-Miller-index substrate structures. It consists of a hexagonal arrangement of Rh atoms sandwiched between two layers of oxygen atoms, differing just slightly by their lattice constant (3.07/3.09 Å, 3.02 Å and 3.04 Å for Rh(100), Rh(111) and Rh(110), respectively [10, 11, 13]) and by a minimal distortion in the case of Rh(100). Illustrating these findings, Supplementary Fig. 2 shows atomic ball models of the metallic and respective trilayer oxide structures for Rh(100) (Supplementary Fig. 2a), Rh(111) (Supplementary Fig. 2b) and Rh(110) (Supplementary Fig. 2c) and the corresponding surface unit cells, constructed from literature data [10, 11, 13].

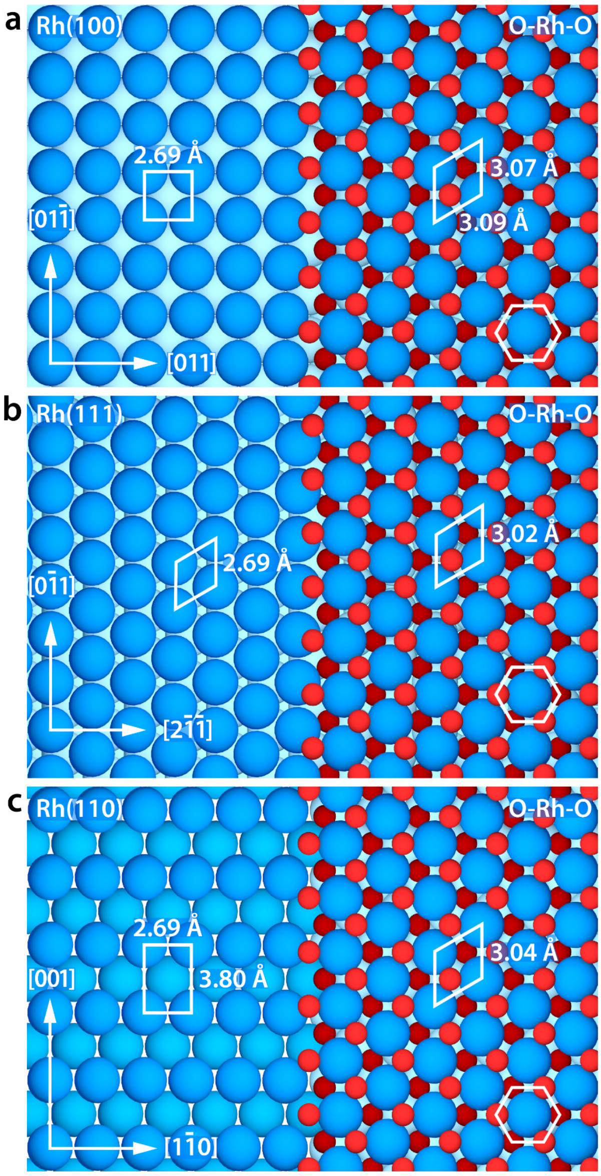

Supplementary Figure 2. Atomic ball models of the trilayer oxide structures on (a) Rh(100), (b) Rh(111) and (c) Rh(110), in comparison to the corresponding metallic surface structures and their respective surface unit cells. Based on data in Refs. [10, 11, 13].

A typical Rh $3d_{5/2}$ XPS spectrum of the Rh surface oxide obtained by scanning photoelectron microscopy (SPEM) in the present experiments reveals three components (Fig. 2 in the main article): (i) a bulk (metallic) component; (ii) a component shifted by about 0.3 eV to lower binding energies respective to the bulk, which is attributed to the interface Rh layer between the metal bulk and surface oxide and (iii) a component shifted by about 0.8 eV to higher binding energies, which corresponds to the Rh layer in the surface oxide. Supplementary Table 1 summarises typical binding energies from literature in comparison to the present data. In addition to the synchrotron-based XPS, we also performed lab-XPS measurements in the course of the catalytic experiments. The lower spectral resolution in our lab-XPS setup unfortunately impedes differentiation between the metallic and interface components, therefore the corresponding spectra (Fig. 3 in the main article) just show two components. Nevertheless, distilling of the surface oxide contribution is still possible, thus proving the presence of surface oxide (the oxidation procedure was the same as in the synchrotron-based experiments). All spectra in the present work were deconvoluted using a pseudo-Voigt lineshape [14] in combination with a Shirley background [15]. The energy scales were calibrated against the energy of the Au $4f_{7/2}$ peak with a binding energy of 84.0 eV.

Supplementary Table 1. Binding energies (in eV) of Rh $3d_{5/2}$ components for the Rh trilayer surface oxide on different Rh(hkl) substrates. The conditions for surface oxide formation and values for the Rh_2O_3 and RhO_2 bulk oxides are listed for comparison.

			Binding energy [eV]			
Substrate	T [K]	p_{O_2} [mbar]	Bulk	Oxide	Interface	Ref.
Rh(111)	650	2×10^{-4}	307.2	308.0	307.0	S11
Rh(110)	750	$> 10^{-4}$	307.2	307.9	306.9	S13
Rh(100)	700	5×10^{-5}	307.2	308.0	306.9	S10
Rh(553)	823	1×10^{-3}	307.2	308.0	307.0	S46
Rh(poly)	623	2.5×10^{-4}	307.3	308.1	307.1	this work
Rh_2O_3				308.3		S47
RhO_2				308.6		S47

The surface oxidation of rhodium is known to start at the lower coordinated Rh atoms at atomic steps, especially at kink sites via the formation of RhO_2 mono- and dimers [16]. Subsequently, a chain of oxygen atoms at the step edges and Rh ridges is formed [17, 18]. The oxidation process continues via diffusion of oxygen under the step edges [11, 18, 19], which poses a kinetic hindrance after reaching a penetration depth of several atomic layers [18, 19]. This results in patches of oxide extending into the step edges but not covering the whole terrace, even after continued oxygen exposure. Furthermore, it was shown that the atomic structure of the step edge determines the kinetic barrier for oxygen penetration [18] and thus also the width and/or shape of the oxide patches. As a result, the ratio of oxide covered and metallic areas varies with the atomic structure of the surfaces. As the atomic structures of the trilayer oxide are identical for all low-Miller-index surfaces (and as a result also for the terraces of any high-Miller-index surface), the XPS peak positions and relative intensities are nearly identical (Supplementary Tab. 1). The observed variation in relative peak intensity between the studied surfaces can thus only stem from the varying ratios of oxide covered and metallic areas. Therefore, evaluation of the XPS spectra for areas of different crystallographic orientation (i.e. different domains of a polycrystalline sample) oxidised at identical conditions allow comparison of the extent of the Rh surface oxide formation for surfaces of differing atomic structures.

Supplementary Note 3: Introduction of SDP and SEP parameters

As detailed in Supplementary Note 2, the surface oxidation of rhodium is essentially determined by the presence of atomic steps (and their density) and the atomic structure of the step edges [17-19]. In order to enable quantitative interpretation of the obtained data on surface oxidation, the studied surfaces have to be numerically characterised with respect to these features. The Terrace-Step-Kink (TSK) model [20, 21] provides a suitable framework for interpretation of the atomic structure of a given surface and serves as a basis for the proposed numerical description. Previously used criteria (e.g. geometric step and kink densities [22, 23] or a weighted average number of nearest neighbours [24, 25]) were insufficient for the current purpose, however. The insufficiency results from neglecting essential features such as the particular structure of a terrace or its step edge (in the case of the geometric step and kink densities, respectively) or because the relative contributions of step density and step edge structure (in the case of an average number of nearest neighbours) are not accurately considered. As it is important to consider both the step density and step edge structure, we have developed a set of parameters describing these features, namely the step density parameter (SDP) and the step edge parameter (SEP), respectively. Both parameters are based on the concept of the surface free energy $\gamma_{(hkl)}$, which has previously been used e.g. for calculating equilibrium shapes of crystals according to the Wulff theorem [26-28] or to study the structural effects of surface defects [29, 30]. A first approximation of the surface free energy is given by calculating the average number of broken bonds for a given surface [31]. For fcc crystals, such as rhodium, the surface free energy $\gamma_{(hkl)}$ of the (hkl) surface according to the broken bond model is calculated in Ref. [32]. By normalising the results to the (111) surface, yielding the relative surface free energy $\gamma_{rel,(hkl)}$, one circumvents the need for physical properties in the calculation. Equation 1 gives the result of such normalisation for a given (hkl) surface, where *h*, *k* and *l* denote the corresponding Miller indices.

$$\gamma_{\mathrm{rel,(hkl)}} = \frac{\gamma_{\mathrm{(hkl)}}}{\gamma_{(111)}} = \frac{\sqrt{3}}{3\sqrt{h^2+k^2+l^2}} \begin{pmatrix} h \\ k \\ l \end{pmatrix} * \begin{pmatrix} 2 \\ 1 \\ 0 \end{pmatrix} \tag{1}$$

Both SDP and SEP are calculated by first constructing a surface, which is equivalent to the described surface in one aspect, but simplifies other aspects. The equivalent surface for calculation of the SDP is characterised by having the exactly same terrace structure and width, but simplified non-kinked step edges (i.e. the steps are of (111)-, (110)- or (100)-type). Calculating $\gamma_{rel,(hkl)}$ according to Eq. 1, using the Miller indices of this first equivalent surface, yields the SDP. For the SEP, in turn, the equivalent surface is constructed by extracting the step edge rows from the original surface and composing a new surface from these rows (i.e. pushing these rows together). This yields a surface which exactly describes the step edge structure, but totally neglects the terraces present in the original surface. The SEP is obtained by calculating $\gamma_{rel,(hkl)}$ according to Eq. 1 using the Miller indices of this second equivalent surface.

The process is exemplarily shown for Rh(13 9 1) in Supplementary Fig. 3. Searching for the equivalent surface for calculation of the SDP yields the Rh(12 8 0) surface, which also has

(110)-type terraces of similar width, but plain (111)-type step edges. This results in an SDP of 1.281 for Rh(13 9 1) by calculating $\gamma_{rel,(hkl)}$ for Rh(12 8 0) using Eq. 1. Constructing the equivalent surface for the SEP produces the Rh(5 1 1) surface, corresponding to an SEP of 1.222 for Rh(13 9 1) by calculating $\gamma_{rel,(hkl)}$ for Rh(5 1 1) using Eq. 1.

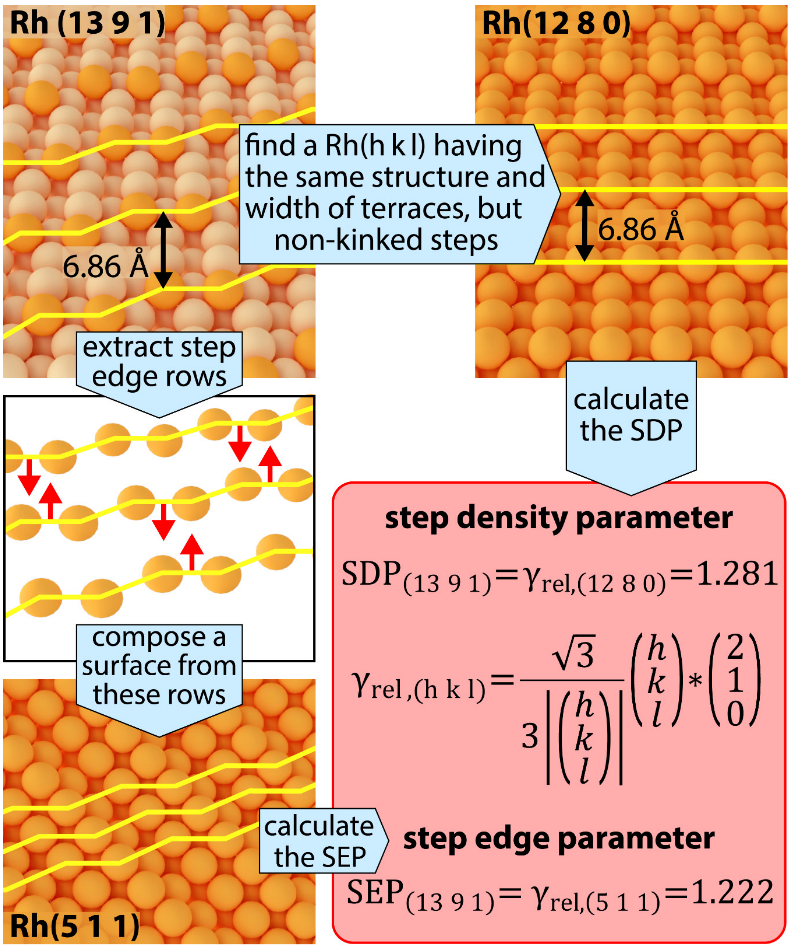

$$\gamma_{rel,(h\,k\,l)} = \frac{\sqrt{3}}{3\left|\begin{pmatrix} h \\ k \\ l \end{pmatrix}\right|} \begin{pmatrix} h \\ k \\ l \end{pmatrix} * \begin{pmatrix} 2 \\ 1 \\ 0 \end{pmatrix}$$

Supplementary Figure 3. Step density (SDP) and step edge (SEP) parameters for the Rh(13 9 1) surface. The SDP is calculated as the relative surface free energy $\gamma_{h,(hkl)}$ of a surface with the same terrace structure and width as Rh(13 9 1), but having non-kinked step edges. In the present case a surface constructed in this way results in a Rh(12 8 0) structure. The SEP is calculated as the relative surface free energy of a surface consisting only of the step edge rows extracted from the Rh(13 9 1) surface (resulting in a Rh(5 1 1) structure in the present case).

The above procedure has been applied to the whole set of domains studied in the SPEM and PEEM experiments and their structures are shown as atomic ball models in Supplementary Fig. 1c. Supplementary Table 2 lists the Miller indices and the relevant equivalent surfaces for calculation of SDP and SEP, as well as the calculation results for the above-mentioned set of ten domains.

Supplementary Table 2. Equivalent surfaces and calculated atomic structure parameters for the set of ten different surface structures shown in Supplementary Fig. 1 and studied in detail in the SPEM and PEEM experiments (Equiv. surf.: equivalent surface, SDP: step density parameter, SEP: step edge parameter).

		Step density		Step edge	
Number	Miller indices	Equiv. surf.	SDP	Equiv. surf.	SEP
1	(11 11 7)	(11 11 7)	1.117	(1 1 0)	1.225
2	(13 9 1)	(12 8 0)	1.281	(5 1 1)	1.222
3	(15 10 9)	(14 9 9)	1.129	(6 1 0)	1.234
4	(18 1 1)	(18 1 1)	1.183	(1 1 1)	1.000
5	(11 7 3)	(8 4 0)	1.290	(7 3 3)	1.199
6	(2 1 1)	(2 1 1)	1.179	(1 0 0)	1.155
7	(12 12 5)	(12 12 5)	1.175	(1 1 1)	1.000
8	(17 10 2)	(15 8 0)	1.291	(9 2 2)	1.224
9	(17 10 5)	(12 5 5)	1.202	(2 2 1)	1.154
10	(19 5 0)	(19 5 0)	1.264	(1 1 0)	1.225

Supplementary Note 4: O *1s* spectra of the oxidised Rh foil

The characteristic fingerprint in the Rh *3d* XPS spectra (Fig. 2 in the main article) allows reliably identifying the RhO_x formed in the present study as the trilayer oxide described in Supplementary Note 2. However, the spectroscopic evidence can be complemented by O *1s* spectra. Supplementary Fig. 4 exemplarily shows such spectra after oxidation in molecular oxygen (T = 623 K, $p_{O_2} = 2.5 \times 10^{-4}$ mbar, t = 90 min) for the Rh (11 11 7) domain (domain number 1 in Supplementary Fig. 1) and the Rh(13 9 1) domain (domain number 2 in Supplementary Fig. 1). As described in literature [10, 11, 13], a typical O *1s* spectrum of Rh trilayer oxide reveals two components: (i) a component corresponding to the topmost oxygen atoms (i.e. the surface oxygen layer) and (ii) a component corresponding to the oxygen atoms sandwiched between the first two Rh layers (i.e. the ordered subsurface oxygen layer), the latter shifted by about 1.1 eV to higher binding energies. As described in the main text and in Supplementary Note 2, the trilayer oxide forms stripes, but does not fully cover wider terraces due to kinetic limitations at the present conditions. As a result, some metallic stripes remain covered by adsorbed oxygen, which contributes to the O *1s* component of the topmost atoms in the trilayer oxide.

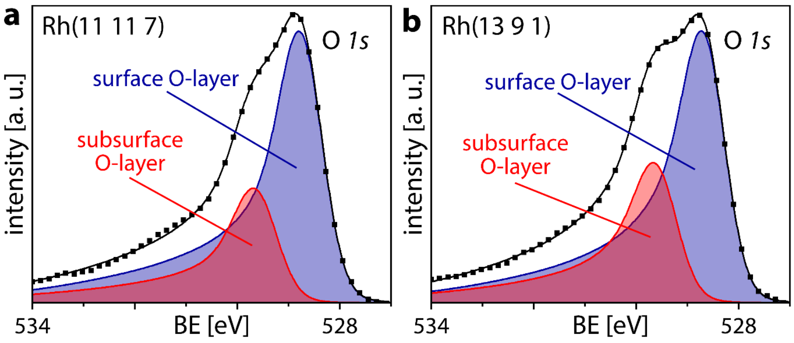

Supplementary Figure 4. O *1s* spectra of the oxidised Rh foil: (a) O *1s* spectrum measured locally for the Rh(11 11 7) domain after oxidation in O_2 (T = 623 K, $p_{O_2} = 2.5 \times 10^{-4}$ mbar, t = 90 min), the corresponding Rh *3d* spectrum is shown in Fig. 2c in the main article; (b) the same as in (a), but for the Rh(13 9 1) domain, the corresponding Rh *3d* spectrum is shown in Fig. 2d in the main article. Squares: measured values; solid lines: sum of the deconvoluted components.

The peak positions match the literature reports on trilayer surface oxide well [10, 11, 13]. Certain deviations of the peak area ratios between the two components can be traced back to the presence of adsorbed oxygen on those parts of terraces which remain uncovered by the oxide stripes in the present experiments.

Supplementary Note 5: Visualisation of catalytic H_2 oxidation by PEEM

Catalytic hydrogen oxidation on platinum group metal surfaces, such as Rh, proceeds via the Langmuir-Hinshelwood mechanism and the reaction rate is thus determined primarily by the competitive dissociative co-adsorption of hydrogen and oxygen [33-35]. At low H_2/O_2 partial pressure ratios, the surface is saturated by oxygen, inhibiting the adsorption of hydrogen and the system is in an inactive steady state (so-called oxygen poisoning [17, 36], corresponding ball model in Supplementary Fig. 5a). Upon increasing the H_2/O_2 ratio, small surface areas with adsorbed H atoms can be observed, where OH islands are formed [17, 36]. The surface density is higher for OH species than for oxygen [17], creating free sites for hydrogen adsorption and H_2O is produced. These catalytically active areas spatially extend over the whole surface via a reaction front, with hydrogen adsorption being blocked ahead and occurring behind it. Due to the high reaction rate, the surface behind the front exhibits both low hydrogen and low oxygen coverage. Impinging hydrogen and oxygen react off, forming immediately desorbing H_2O and the system remains in the active steady state (corresponding ball model in Supplementary Fig. 5a).

Using photoemission electron microscopy (PEEM), the above-mentioned states can be identified in situ during the ongoing reaction. In PEEM, the image intensity is determined by the local work function of the imaged surface, i.e. by the coverage of reactants. The reaction rate in turn also depends on the surface coverage of the reactants, dark image contrast (high work function, oxygen covered surface) thus corresponds to the catalytically inactive state. In turn, areas of bright image contrast (low work function, nearly adsorbate-free surface) are corresponding to high catalytic activity, thus the PEEM image brightness reflects the local catalytic activity. Proven quantitatively by averaging over the whole field of view [33], the relationship between image brightness and catalytic activity can be scaled down to each single pixel in the PEEM image. Resulting from the spatial distribution of the work function over the sample surface, the local PEEM intensity reflects the local H_2O production rate [37]. This procedural method known as local kinetics by imaging approach, was recently successfully applied to different model systems and different length scales [6, 8, 25, 38].

Supplementary Fig. 5a, showing schematically drawn PEEM images, explains this approach for the present PEEM experiments with cyclewise variation of p_{H_2} at constant T and p_{O_2}. Starting from the inactive oxygen covered surface (frame 1) and increasing p_{H_2}, a kinetic transition, accompanied by spreading reaction fronts (frame 2) to the catalytically active state (frame 3) takes place at a certain $p_{H_2} = \tau_A$. Upon decreasing the hydrogen partial pressure back from the active state (frame 4, identical to frame 3), the reverse kinetic transition takes place at a $p_{H_2} = \tau_B$ (frame 5) to the catalytically inactive state (frame 6). The value of τ_B appears to be significantly lower than τ_A, i.e. a hysteresis is observed (Supplementary Fig. 5b).

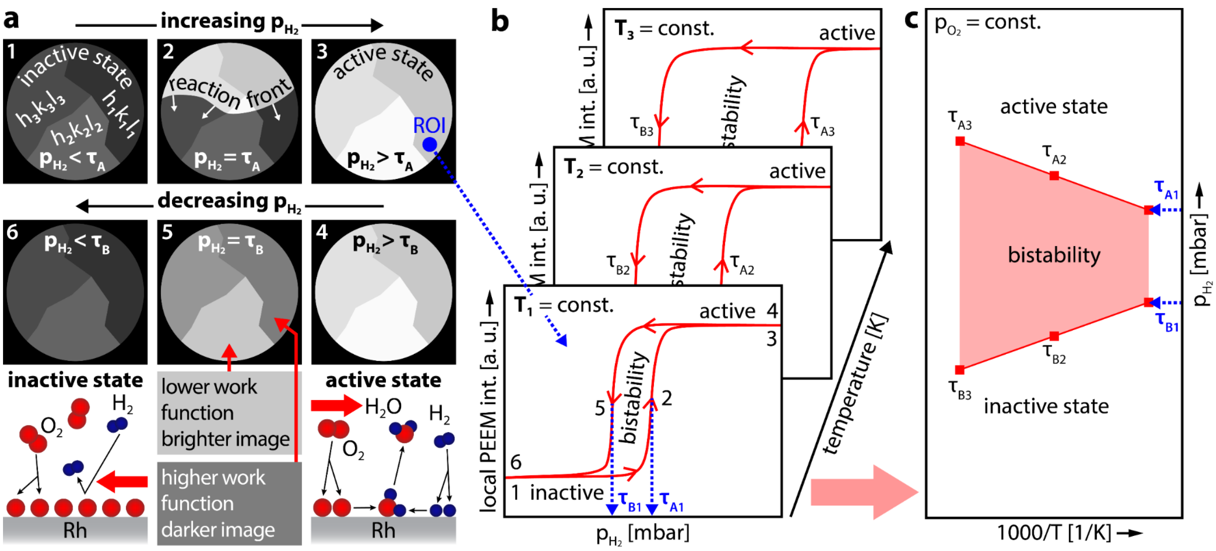

Supplementary Figure 5. Principle of the catalytic PEEM experiments: (a) frames 1 to 6 show schematic PEEM images upon cyclewise variation of p_{H_2} at constant T and p_{O_2}. A graphics of the catalytically inactive and active states is shown below; (b) schematic hysteresis curves resulting from the local PEEM image brightness in a selected region of interest (ROI) as a function of the hydrogen partial pressure; (c) kinetic phase diagram resulting from the hysteresis curves and summarising the kinetic transition points at different temperatures, showing the states of low and high catalytic activity and bistability.

This hysteresis-like behaviour is a result of the unequal adsorption properties of the reactants [33] and is reflected in the local PEEM image brightness plotted against the hydrogen partial pressure (Supplementary Fig. 5b). The resulting plots are typical for a bistable character of the reaction, i.e. two steady states (high and low catalytic activity) can exist at the same set of external parameters and the actual state is determined solely by the prehistory of the system [39].

Upon repeating the described experiment at different temperatures, a set of transition points can be collected and summarised in a kinetic phase diagram, as shown in Supplementary Figs. 5b and 5c. A detailed discussion concerning the term kinetic phase diagram can be found elsewhere [40-43]. Such diagrams show areas of high and low catalytic activity and bistability at one glance and allow comparison of the performance of different catalysts, as well as the prediction of temperatures and partial pressures of kinetic transitions.

Supplementary Note 6: Partial reduction of surface oxide during H_2 oxidation

The Rh surface oxide can be reduced during exposure to a reducing atmosphere [44, 45]. Therefore, the surface oxide formed in our experiments will be inevitably reduced to some extent in the catalytically active steady state. During the cyclewise variation of the H_2 pressure in the PEEM experiments on the oxidised Rh surface, the reduction may start immediately after the increasing p_{H_2} reaches τ_A (Supplementary Fig. 5). This has, of course, an effect on the hysteresis loop "on the way back", i.e. during the following decrease of p_{H_2}, and this also influences the position of the resulting τ_B transition. To shed light on this effect, we performed

experiments consisting of serial cycles of increasing and decreasing the hydrogen partial pressure, one right after the other.

Supplementary Fig. 6 exemplarily shows the hysteresis curves obtained in such experiments during cyclewise variation of p_{H_2} from 1.0 x 10^{-7} to 5.0 x 10^{-6} mbar and vice versa, at a constant temperature of 463 K and constant p_{O_2} of 7.7 x 10^{-7} mbar. The surface region for which the PEEM intensity curves were registered corresponds to the one analysed in Fig. 3b in the main article.

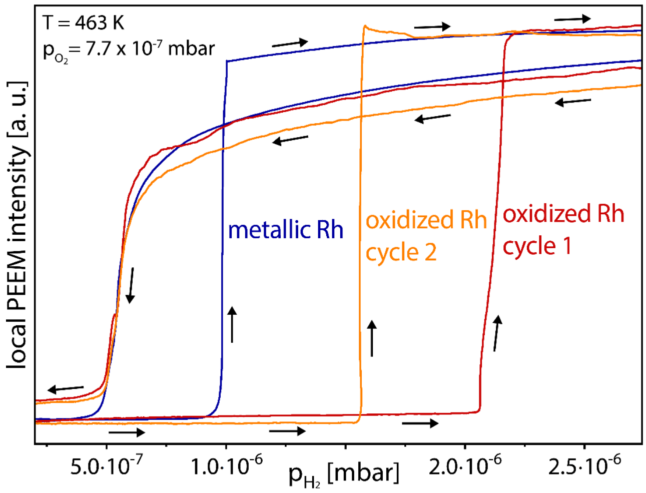

Supplementary Figure 6. Hysteresis curves of the local PEEM intensity registered in serial ("one right after the other") cyclewise variations of p_{H_2} at constant p_{O_2} = 7.7 x 10^{-7} mbar and T = 463 K. In addition, the hysteresis curve for the metallic Rh surface is shown for comparison (which shows no changes when repeated).

The experiments reveal that indeed reduction of the surface oxide takes place during the catalytically active period. However, reduction is only partial in the first cycle, since the kinetic transition τ_A during the second cycle still occurs at significantly higher hydrogen pressure than for the metallic surface. It is therefore justified to assume that surface oxide survives at least one hysteresis cycle, i.e. the results summarised in the phase diagrams (Fig. 3 in the main article) and velocity map (Fig. 4 in the main article) definitely concern the Rh surface oxide.

Supplementary References

[1] Dingley, D. J., Baba-Kishi, K. Z. & Randle V. *Atlas of Backscattering Kikuchi Diffraction Patterns* (IOP Publishing, 1995).

[2] Baba-Kishi, K. Z. Review Electron backscatter Kikuchi diffraction in the scanning electron microscope for crystallographic analysis. *J. Mater. Sci.* **37,** 1715–1746 (2002).

[3] Dingley, D. J. & Randle, V. Microtexture determination by electron back-scatter diffraction. *J. Mater. Sci.* **27,** 4545–4566 (1992).

[4] Schwartz, A. J., Kumar, M., Adams, B. L. & Field, D. P. (eds.) *Electron Backscatter Diffraction in Materials Science* (Springer US, 2009).

[5] König, U. & Davepon, B. Microstructure of polycrystalline Ti and its micro-electrochemical properties by means of electron-backscattering diffraction (EBSD). *Electrochim. Acta.* **47,** 149–160 (2001).

[6] Vogel, D. *et al.* The Role of Defects in the Local Reaction Kinetics of CO Oxidation on Low-Index Pd Surfaces. *J. Phys. Chem. C* **117,** 12054–12060 (2013).

[7] Weatherup, R. S. *et al.* In Situ Graphene Growth Dynamics on Polycrystalline Catalyst Foils. *Nano Lett.* **16,** 6196–6206 (2016).

[8] Suchorski, Y. *et al.* Visualizing catalyst heterogeneity by a multifrequential oscillating reaction. *Nat. Commun.* **9,** 600:1–6 (2018).

[9] Humphreys, F. J. Grain and subgrain characterisation by electron backscatter diffraction. *J. Mater. Sci.* **36,** 3833–3854 (2001).

[10] Gustafson, J. *et al.* Structure of a thin oxide film on Rh(100). *Phys. Rev. B* **71,** 115442-1–9 (2005).

[11] Gustafson, J. *et al.* Self-Limited Growth of a Thin Oxide Layer on Rh(111). *Phys. Rev. Lett.* **92,** 126102-1–4 (2004).

[12] Blomberg, S. *et al.* Structure of the Rh_2O_3(0001) surface. *Surf. Sci.* **606,** 1416–1421 (2012).

[13] Dri, C. *et al.* Initial oxidation of the Rh(110) surface: Ordered adsorption and surface oxide structures. *J. Chem. Phys.* **125,** 094701-1–9 (2006).

[14] Evans, S. Curve Synthesis and Optimization Procedures for X-ray Photoelectron Spectroscopy. *Surf. Interface. Anal.* **17,** 85–93 (1991).

[15] Shirley, D. A. High-Resolution X-Ray Photoemission Spectrum of the Valence Bands of Gold. *Phys. Rev. B* **5,** 4709–4714 (1972).

[16] Mittendorfer, F. *et al.* Oxygen-Stabilized Rh Adatoms: 0D Oxides on a Vicinal Surface. *J. Phys. Chem. Lett.* **2,** 2747–2751 (2011).

[17] Africh, C. *et al.* Two-Step Reaction on a Strained, Nanoscale Segmented Surface. *Phys. Rev. Lett.* **93,** 126104-1–4 (2004).

[18] Klikovits, J. *et al.* Step-Orientation-Dependent Oxidation: From 1D to 2D Oxides. *Phys. Rev. Lett.* **101,** 266104-1–4 (2008).

[19] Farber, R. G. *et al.* The Quest for Stability: Structural Dependence of Rh(111) on Oxygen Coverage at Elevated Temperature. *J. Phys. Chem. C* **121,** 10470–10475 (2017).

[20] Kossel, W. Zur Theorie des Kristallwachstums. *Nachr. Ges. Wiss. Göttingen, Math.-Phys. Klasse* **1927,** 135–143 (1927).

[21] Stranski, I. N. Zur Theorie des Kristallwachstums. *Z. Phys. Chem.* **136U,** 259–278 (1928).

[22] Gruber, E. E. & Mullins W. W. On the theory of anisotropy of crystalline surface tension. J. *Phys. Chem. Solids* **28,** 875–887 (1967).

[23] Böller, B., Durner K. M. & Wintterlin J. The active sites of a working Fischer–Tropsch catalyst revealed by operando scanning tunnelling microscopy. *Nat. Catal.* **2,** 1027–1034 (2019).

[24] Calle-Vallejo, F., Martinez, J. I., Garcia-Lastra, J. M., Sautet, P. & Loffreda, D. Fast Prediction of Adsorption Properties for Platinum Nanocatalysts with Generalized Coordination Numbers. *Angew. Chem. Int. Edit.* **53,** 8316–8319 (2014).

[25] Suchorski, Y. *et al.* CO Oxidation on Stepped Rh Surfaces: µm-Scale Versus Nanoscale. *Cat. Lett.* **150,** 605–612 (2020).

[26] Wulff, G. XXV. Zur Frage der Geschwindigkeit des Wachsthums und der Auflösung der Krystallflächen. *Z. Kristallogr.* **34,** 449–530 (1901).

[27] v. Laue, M. XXV. Der Wulffsche Satz für die Gleichgewichtsform von Kristallen. *Z. Kristallogr.* **105,** 124–133 (1943).

[28] Barmparis, G. D., Lodziana, Z., Lopez, N. & Remediakis I. N. Nanoparticle shapes by using Wulff constructions and first-principles calculations. *Beilstein J. Nanotech.* **6,** 361–368 (2015).

[29] Bonzel, H. P. Surface Morphologies: Transient and Equilibrium Shapes. *Interface. Sci.* **8,** 21–34 (2001).

[30] Grochola, G., du Plessis, J., Snook, I. K. & Russo, S. P. On the computational calculation of adatom, vacancy and early stage surface nucleation island free energies on the (1 1 1) copper surface. *Surf. Sci.* **591,** 32–37 (2005).

[31] Stranski, I. N. Forms of equilibrium of crystals. *Discuss. Faraday Soc.* **5,** 13–21 (1949).

[32] Mackenzie, J. K., Moore, A. J. W. & Nicholas, J. F. Bonds broken at atomically flat crystal surfaces—I: Face-centred and body-centred cubic crystals. *J. Phys. Chem. Solids* **23,** 185–196 (1962).

[33] Schaak, A. & Imbhil, R. Bistability and formation of low work function areas in the O_2+H_2 reaction on a Rh(111) surface. *J. Chem. Phys.* **113,** 9822–9829 (2000).

[34] Ertl, G. Reactions at Surfaces: From Atoms to Complexity (Nobel Lecture). *Angew. Chem. Int. Edit.* **47,** 3524–3535 (2008) and references therein.

[35] Yates, J. T. Jr., Thiel, P. A. & Weinberg, W. H. Reactions at Surfaces: The catalytic reaction between adsorbed oxygen and hydrogen on Rh(111). *Surf. Sci.* **82,** 45–68 (1979).

[36] Sachs, C., Hildebrand, M., Völkening, S., Wintterlin, J. & Ertl, G. Reaction fronts in the oxidation of hydrogen on Pt(111): Scanning tunneling microscopy experiments and reaction–diffusion modeling. *J. Chem. Phys.* **116,** 5759–5773 (2002).

[37] Suchorski, Y. & Rupprechter, G. Local reaction kinetics by imaging. *Surf. Sci.* **643,** 52–58 (2016).

[38] Datler, M. *et al.* Hydrogen Oxidation on Stepped Rh Surfaces: µm-Scale versus Nanoscale. *Cat. Lett.* **146,** 1867–1847 (2016).

[39] Vogel, D. *et al.* Mapping the local reaction kinetics by PEEM: CO oxidation on individual (100)-type grains of Pt foil. *Surf. Sci.* **605,** 1999–2005 (2011).

[40] Zhdanov, V. P. & Kasemo B. Kinetic phase transitions in simple reactions on solid surfaces. *Surf. Sci. Rep.* **20,** 113–189 (1994).

[41] Schlögl, F. Chemical reaction models for non-equilibrium phase transitions. *Z. Phys.* **253,** 147–161 (1972).

[42] Schlögl, F. What Can we Learn about Dissipative Structures by Model Reactions. *Berich. Bunsen Gesell.* **84,** 351–357 (1980).

[43] Ehsais, M. *et al.* A reactive phase diagram of CO oxidation on Pd(110): Steady and oscillatory states. *J. Chem. Phys.* **98,** 9177–9184 (1993).

[44] Lundgren, E. *et al.* The surface oxide as a source of oxygen on Rh(1 1 1). *J. Electron Spectrosc.* **144-147,** 364–372 (2005).

[45] Dudin, P. *et al.* Initial Oxidation of a Rh(110) Surface Using Atomic or Molecular Oxygen and Reduction of the Surface Oxide by Hydrogen. *J. Phys. Chem. B* **109,** 13649–13655 (2005).

[46] Gustafson, J. *et al.* Oxygen-induced step bunching and faceting of Rh(553): Experiment and ab initio calculations. *Phys. Rev. B* **74,** 035401-1–7 (2006).

[47] Abe, Y., Kato, K., Kawamura, M. & Sasaki, K. Rhodium and Rhodium Oxide Thin Films Characterized by XPS. *Surf. Sci. Spec*. **8**, 117–125 (2001).
