# Supplementary material for: How the anisotropy of surface oxide formation influences the transient activity of a surface reaction
Source: Nat Commun. 2021 Jan 4;12:69. doi: 10.1038/s41467-020-20377-9 (PMC7782819; doi:10.1038/s41467-020-20377-9)
Supplement: Supplementary file 2 — Reporting Summary [file 41467_2020_20377_MOESM2_ESM.pdf]

nature research

Corresponding author(s): NCOMMS-20-35336-T

Last updated by author(s): Oct 30, 2020

Reporting Summary

Nature Research wishes to improve the reproducibility of the work that we publish. This form provides structure for consistency and transparency in reporting. For further information on Nature Research policies, see our Editorial Policies and the Editorial Policy Checklist.

Statistics

For all statistical analyses, confirm that the following items are present in the figure legend, table legend, main text, or Methods section.

n/a	Confirmed	
☐	☒	The exact sample size (n) for each experimental group/condition, given as a discrete number and unit of measurement
☐	☒	A statement on whether measurements were taken from distinct samples or whether the same sample was measured repeatedly
☒	☐	The statistical test(s) used AND whether they are one- or two-sided<br>*Only common tests should be described solely by name; describe more complex techniques in the Methods section.*
☐	☒	A description of all covariates tested
☒	☐	A description of any assumptions or corrections, such as tests of normality and adjustment for multiple comparisons
☒	☐	A full description of the statistical parameters including central tendency (e.g. means) or other basic estimates (e.g. regression coefficient) AND variation (e.g. standard deviation) or associated estimates of uncertainty (e.g. confidence intervals)
☒	☐	For null hypothesis testing, the test statistic (e.g. F, t, r) with confidence intervals, effect sizes, degrees of freedom and P value noted<br>*Give P values as exact values whenever suitable.*
☒	☐	For Bayesian analysis, information on the choice of priors and Markov chain Monte Carlo settings
☒	☐	For hierarchical and complex designs, identification of the appropriate level for tests and full reporting of outcomes
☒	☐	Estimates of effect sizes (e.g. Cohen's d, Pearson's r), indicating how they were calculated

Our web collection on statistics for biologists contains articles on many of the points above.

Software and code

Policy information about availability of computer code

Data collection	Custom acquisition software developed at the ESCA microscopy beamline at ELETTRA, Hokawo V3.0, MKS Instruments EasyView V5.76.0.4000, SpecsLab Prodigy V4.60.2
Data analysis	CasaXPS V2.3.22PR1.0, Icy V2.0.3.0, Igor Pro V6.37, OriginPro 2020 V9.7.0.185

For manuscripts utilizing custom algorithms or software that are central to the research but not yet described in published literature, software must be made available to editors and reviewers. We strongly encourage code deposition in a community repository (e.g. GitHub). See the Nature Research guidelines for submitting code & software for further information.

Data

Policy information about availability of data

All manuscripts must include a data availability statement. This statement should provide the following information, where applicable:

- Accession codes, unique identifiers, or web links for publicly available datasets
- A list of figures that have associated raw data
- A description of any restrictions on data availability

The data that support the findings of this study are available from the corresponding author upon reasonable request.

Field-specific reporting

Ecological, evolutionary & environmental sciences study design

All studies must disclose on these points even when the disclosure is negative.

Study description	To reveal the anisotropy of surface oxidation as well as its effect on hydrogen oxidation, ten crystallographically different Rh surfaces were examined by two combined spatio-temporal surface-imaging techniques. Scanning photoelectron microscopy (SPEM) and photoemission electron microscopy (PEEM), which allow local material analysis and visualising ongoing reactions on a µm-scale, respectively, were applied to polycrystalline Rh, representing a library of well-defined high-Miller-index surface structures.
Research sample	Polycrystalline Rhodium foil
Sampling strategy	One sample. Size (10 x 12 mm^2) compatible with experimental setup (sample holders)
Data collection	M.Stöger-Pollach performed the EBSD characterisation. M.Amati, L.Gregoratti, G.Rupprechter, P.Winkler, J.Zeininger and P.Zoller performed the SPEM measurements. P.Winkler and J.Zeininger performed the PEEM measurements. G.Rupprechter and Y.Suchorski supervised the experimental work and were involved in the analysis of the experimental data. G.Rupprechter, Y.Suchorski, P.Winkler and J.Zeininger prepared the manuscript.
Timing and spatial scale	Data taken between and Jan 2019 and Sept 2020, including two synchrotron beamtimes.
Data exclusions	none
Reproducibility	Confirmed by repeated measurments.
Randomization	*Describe how samples/organisms/participants were allocated into groups. If allocation was not random, describe how covariates were controlled. If this is not relevant to your study, explain why.*
Blinding	n.a.

Did the study involve field work? ☐ Yes ☒ No

Reporting for specific materials, systems and methods

We require information from authors about some types of materials, experimental systems and methods used in many studies. Here, indicate whether each material, system or method listed is relevant to your study. If you are not sure if a list item applies to your research, read the appropriate section before selecting a response.

Materials & experimental systems

n/a	Involved in the study
☒	☐ Antibodies
☒	☐ Eukaryotic cell lines
☒	☐ Palaeontology and archaeology
☒	☐ Animals and other organisms
☒	☐ Human research participants
☒	☐ Clinical data
☒	☐ Dual use research of concern

Methods

n/a	Involved in the study
☒	☐ ChIP-seq
☒	☐ Flow cytometry
☒	☐ MRI-based neuroimaging
